# Risk prediction models for malnutrition in patients with cancer: a systematic review

**DOI:** 10.3389/fnut.2026.1900944

**Published:** 2026-08-06

**Authors:** Kaiyao Jiang, Junling Pan, Yuping Zhang

**Affiliations:** Department of Hepatobiliary and Pancreatic Surgery Ward Two, Jinhua Municipal Central Hospital, Jinhua, China

**Keywords:** cancer, malnutrition, oncology, risk prediction model, systematic review

## Abstract

**Background:**

At present, many nutritional risk prediction models have been developed for cancer patients, but there is still uncertainty regarding the methodological quality and clinical applicability of these models.

**Objective:**

To systematically review and critically appraise existing risk prediction models for malnutrition in cancer patients.

**Methods:**

The PubMed, EBSCO, Medline, Web of Science, Scopus and Cochrane Library databases were systematically retrieved. The retrieval period was from the establishment of the databases to September 30, 2025. Based on the Predictive Model Bias Risk of Assessment Tool (PROBAST) and Checklist for Critical Appraisal and Data Extraction for Systematic Reviews of Prediction Modeling Studies (CHARMS), two independent authors conducted a rigorous assessment and data extraction of the study.

**Results:**

A total of 6,343 articles were retrieved, ultimately including 16 studies and 42 models. The sample size included in the studies ranged from 120 to 4,487. Models were developed with logistic regression or machine-learning algorithms. The area under the curve (AUC) for the development cohort ranged from 0.745 to 1.000, while for the validation cohort ranged from 0.687 to 0.982. Twelve studies (75.0%) evaluated model calibration using calibration curves, the Hosmer-Lemeshow test, and the Brier score, demonstrating good calibration performance. All risk prediction models have a relatively high risk of bias, primarily due to issues with the study population and analysis domain, two models (12.5%) raised high concern regarding applicability.

**Conclusion:**

Research on prediction models for malnutrition in cancer patients is still in the development stage. The predictive performance of the developed models is generally acceptable, but there are still deficiencies in validation and evaluation.

## Introduction

The latest statistical report on malignant tumors released by the International Agency for Research on Cancer (IARC) shows that the incidence of cancer continues to rise globally, with nearly 20 million new cases each year and a mortality rate as high as 48.5% (1). Malnutrition is widely recognized as the most common complication among cancer patients (2), its causes include increased energy expenditure due to the tumor's high metabolic state, inadequate dietary intake during the perioperative period, and decreased appetite resulting from chemotherapy-induced nausea and vomiting (3). Malnutrition can lead to severe metabolic disorders and frailty in patients, increase the risk of postoperative complications, and even affect the prognosis of patients (4–6). Previous studies have shown that the prevalence of malnutrition among cancer patients is as high as 41% (4). To improve the nutritional status of cancer patients, researchers have developed a series of nutritional intervention measures, including perioperative enteral and parenteral nutritional support, micronutrient supplementation, the use of antiemetic drugs to alleviate the side effects of chemotherapy drugs, and cognitive behavioral therapy (7–9), etc. Although these measures have improved patients' nutritional status to some extent, many patients are still mistakenly considered to be at no risk of malnutrition (10), and consequently, the nutritional needs of this population remain unmet. Therefore, early identification of high risk malnutrition patients and implementation of early nutritional intervention can help patients withstand the impact of cancer treatment. This is an essential component of patient-centered supportive care and cancer treatment management.

To enable early identification of the occurrence and progression of malnutrition, researchers have developed corresponding malnutrition risk prediction models. Risk prediction models, also known as clinical prediction models, combine multiple predictors factors to estimate the presence of specific conditions or the risk of a future event (11). These models are primarily presented in forms such as regression equations, nomograms, or some explorations based on artificial intelligence (12). At present, numerous prediction models for malnutrition in cancer patients have been developed and applied (3, 13, 14), which help healthcare professionals effectively identify the risk of malnutrition, formulate prevention and treatment strategies, and lay the foundation for providing practical and effective preventive measures. However, the quality and applicability of various models remain unknown. There is no consensus on the most effective model for predicting the risk of malnutrition in cancer patients. Moreover, the onset and progression of malnutrition result from the combined effects of multiple predictive factors. Understanding these risk predictive factors is of great significance for better identifying high-risk populations of malnutrition.

Therefore, this study systematically reviewed the performance and characteristics of current models for predicting the risk of malnutrition in cancer patients and evaluated the risk of bias and applicability. The aim is to provide a reference for improving existing models and for the development and validation of new models, thereby laying a foundation for the clinical application of these models.

## Methods

This systematic review protocol was registered on the PROSPERO (registration number: CRD420251169732).

### Search strategy

The PubMed, EBSCO, Medline, Web of Science, Scopus and Cochrane Library databases were systematically retrieved. The search period was from the establishment of the database to September 30, 2025 with a language restriction to English. The search terms included Mesh terms and free-text terms related to prediction models, oncology and malnutrition, such as predicti^*^ model, prediction, risk model risk assessment, risk prediction, risk score, nomogram, forecast model, diagnostic model, prediction tool, neoplasms, neoplas^*^, malignanc^*^, tumor, cancer, malignant, carcinoma, oncology, neoplasms, malnutrition, undernourishment^*^, nutritional deficienc^*^, dystroph^*^, undernutrition, hyponutrition, paratrophy, hypotrophy, malnutrition. Detailed database search strategies are provided in Supplementary materials (Table S1). We constructed an evidence-based question using the PICOTS model recommended by the Cochrane Prognostic Methods Group, as shown in Table 1 (15).

**Table 1:** PICOTS concept.

Item	Connotation
Population	Adult cancer patients
Index Prediction Model	Model for predicting the risk of malnutrition
Comparator	None
Outcome	Malnutrition defined for an effective malnutrition assessment tool
Timing	The entire stage period of cancer patients, including diagnosis, treatment, or recovery
Setting	For healthcare institutions such as hospitals or clinics

### Inclusion and exclusion criteria

Inclusion criteria: (1) Adult cancer patients with a clear pathological diagnosis (cancer stage, type, and treatment method is not restricted) (≥18 years old); (2) Outcome indicators are malnutrition defined by valid malnutrition assessment tools; (3) Research types included case-control studies, cross-sectional studies, and cohort studies in English literature; (4) The research content is the construction and/or verification of a malnutrition risk prediction model. Exclusion criteria: (1) Studies that only analyzed risk factors for malnutrition in cancer patients without establishing prediction models; (2) Studies that only reported the predictive value of a single factor for malnutrition; (3) Literature lacking detailed descriptions of model construction procedures, as well as those involving duplicate publications, poor quality, or incomplete data; (4) Literature classified as reviews, systematic reviews, conference abstracts, research protocols, or commentaries.

### Study selection and data extraction

We use EndNote20 for literature management and filter literature based on title, abstract and full text. A standardized data extraction form was developed based on the Checklist for Critical Appraisal and Data Extraction for Systematic Reviews of Prediction Modeling Studies (16) (CHARMS) checklist to extract information. The extracted content includes: (1) Basic information: first author, year of publication, country, research design, timing of use, data source, subjects, outcome measure and evaluation tools; (2) Model information: sample size and outcome event, methods for selecting predictors, number of candidate predictors, final predictors number and catalogs, missing data and handling methods, model development methods and number, model performance (development set and validation set), model calibration method, cut-off value, internal and/or external validation and model presentation forms, etc. Literature screening and data extraction were independently conducted by two researchers and cross-checked. In case of any disagreement, discussions were held with the third researcher.

### Risk of bias and application assessment

Two researchers independently assessed the risk of bias and applicability of the included studies using the Prediction Model Risk of Bias Assessment Tool (12) (PROBAST). Bias risk assessment encompasses four areas: research subjects, predictors, outcomes, and data analysis, involving a total of 20 signaling questions. Each question should be answered as “yes or probably yes,” “no or probably no” and “not clear.” When all questions within a field are answered as “yes or probably yes,” the risk of bias in that field is relatively low. The response of “no or probably no ” indicates a relatively high risk of bias in this field. When one question is answered as “not clear” while all other questions are answered as “yes or probably yes,” the risk of bias in that area is “not clear.” When the risk of bias in all domains is low, the overall risk of bias in the study is low. If the risk of bias in just one domain is high, the overall risk of bias is high. If the risk of bias in a certain area is “not clear” while the risk of bias in other areas is low, then the overall risk of bias is classified as “not clear.” The applicability assessment involves the first three areas, and the assessment process and standards are similar to those of the risk of bias assessment.

### Data synthesis

Given the significant heterogeneity among different studies in terms of study populations, outcome definitions, and measurement methods, we adopted a narrative approach to qualitatively summarize the results and did not perform a meta-analysis. This study summarized and sorted out the included studies. Firstly, the baseline characteristics such as the research type, subjects, application timing, result definition and measurement methods of each study were summarized. Subsequently, the key elements of the model construction and validation process are listed in detail, namely the sample size and outcome events, the number of candidate variables, the information of missing values and the methods for handling missing values, as well as the final retained predictors, etc. Finally, the model's performance is presented, including discriminatory power, calibration, internal and external validation, and model presentation; a comprehensive evaluation is conducted to assess the applicability and interpretability of the malnutrition prediction model in cancer patients.

## Results

### Study selection

**Figure 1:**
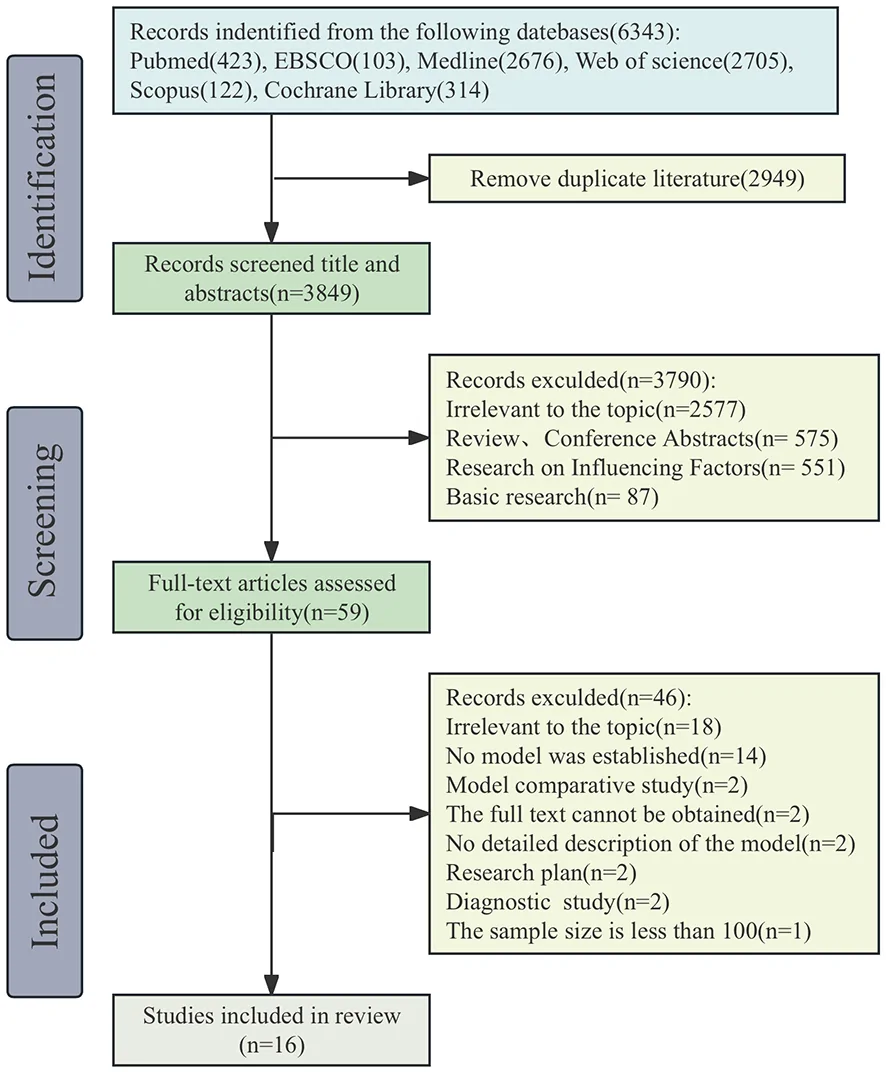
Flowchart of the literature search, screening, and final included studies.

The Preferred Reporting Items for Systematic reviews and Meta Analyses (PRISMA) 2020 (17) flowchart for the study selection process is presented in Figure 1. In total, 6343 records were obtained by searching databases, and 2494 duplicates were excluded. By reading titles and abstracts, 3,790 articles were excluded. Subsequently, after reading the full texts, 43 articles were excluded, resulting in 16 articles for the review (13, 14, 18–31).

### Basic characteristics of the included studies

This review included 16 studies published between 2021 and 2025. Fourteen studies were conducted in China (14, 18–30), South Korea published one study (13), and the United States published one study (31). Ten studies (13, 18, 20–25, 28, 30)were retrospective studies, three studies (14, 19, 29)were multicenter observational cohort studies, and the data were all derived from the Investigation on Nutrition Status and Clinical Outcome of Common Cancers (INSCOC) project (32) (chictr.org.cn:ChiCTR1800020329), two studies (26, 31)for cross-sectional study, One was a single-center retrospective cross-sectional study (27). All the research subjects were cancer patients, with cancer types including gastric cancer, lung cancer, colorectal cancer, cervical cancer and nasopharyngeal cancer, etc. The application scenarios of the predictive model include before surgery, after surgery, during hospitalization, upon admission, at the time of tumor diagnosis, and after radiotherapy for patients. Among the included studies, one study used Malnutrition Universal Screening Tool (MUST) for the diagnosis of malnutrition (13), six used the Global Malnutrition Leadership Initiative Criteria (GLIM) (14, 19, 24, 27–29), six used the Patient Subjective Global Assessment (PG-SGA) scale (18, 20–23, 26), one used the Malnutrition Screening Tool (MST) (31), and two used the NRS2002 assessment tool (25, 30). The detailed characteristics of the included studies are shown in Table 2.

**Table 2:** Basic characteristics of inclusion in the literature.

References	Country	Study design	Timing of use	Data source	Object	Outcome measure	Evaluation tool
Park et al. (13) (2021)	Korea	retrospective study	6 months after gastrectomy	between January 2014 and December 2015 in a tertiary hospital.	After Gastric Cancer Surgery	BMI < 18.5 kg/m2 a	MUST
Yin et al. (14) (2021)	China	observational cohort study	Before the operation	INSCOC	lung cancer	at least one phenotypic criterion and one etiologic criterion is required b	GLIM criteria
Zhang et al. (18) (2022)	China	retrospective cohort study	During hospitalization	in a tertiary hospital from June 2020 to February 2021	hospitalized adult's tumor patients	PG-SGA > 4 points	PG-SGA scale
Yin et al. (19) (2021)	China	observational cohort study	patient admission	INSCOC	not report	at least one phenotypic criterion and one etiologic criterion is required	GLIM criteria
Dai et al. (20) (2023)	China	retrospective case-control study	three-month-postoperative	From January 2019 to December 2021 in a tertiary hospital	The gastric cancer patients treated with laparoscopic radical gastrectomy	PG-SGA ≥ 4 points	PG-SGA scale
Tang et al. (31) (2023)	USA	Cross-sectional	time of diagnosis	January 2019 and January 2021 in a tertiary referral outpatient oncology clinic	Colorectal Cancer Patients	scored ≥2 were deemed high risk for malnutrition	MST f
Yu et al. (21) (2023)	China	retrospective study	postoperative	July 2020 and June 2022 at the cancer center of a tertiary hospital.	FIGO stage IB1-IIA2 CC who underwent PORT/CRT	PG-SGA score ≥ 4	PG-SGA scale
Duan et al. (22) (2024)	China	retrospective study	During hospitalization	from January 2022 to January 2023 of a tertiary hospital	hospitalized cancer patients aged 60+	PG-SGA score ≥ 4	PG-SGA scale
Duan et al. (23) (2024)	China	retrospective study	During hospitalization	Data from January 2022 to January 2023 of a tertiary hospital	hospitalized cancer patients aged 60+	PG-SGA score ≥ 4	PG-SGA scale
Huang et al. (24) (2024)	China	retrospective study	Before the operation	January 2016 to December 2020 in a tertiary hospital	gastric cancer patients without preoperative neoadjuvant chemotherapy or radiotherapy.	One phenotypic criterion.	GLIM criteria
Li et al. (25) (2024)	China	retroactive study	before gastric cancer surgery	January 2016 to December 2019in a tertiary hospital	gastric cancer patients before surgery	NRS 2002 score of ≥ 3	NRS 2002 scale
Wang et al. (26) (2024)	China	Cross-sectional study	not report	in a tertiary public hospital from May 2022 to July 2023	newly diagnosed nasopharyngeal carcinoma	PG-SGA scores ≥ 2	PG-SGA scale
Wang et al. (27) (2024)	China	retrospective cross-sectional study	During hospitalization	between July 1, 2021, and December 31, 2023 in a tertiary hospital	hospitalized patients over 65 years with malignant tumors	at least one phenotypic criterion and one etiologic criterion.	GLIM criteria
Wang et al. (28) (2024)	China	retrospective cohort study	not report	December 1, 2021 to January 31, 2023 in a tertiary hospital	stage I–IV colorectal cancer	at least one phenotypic criterion and one etiologic criterion.	GLIM criteria
Wu et al. (29) (2024)	China	observational cohort study	not report	INSCOC	colorectal cancer patients without weight loss information	meeting one of the following phenotypic criteria	GLIM criteria
Zhu et al. (30) (2025)	China	retrospective study	after radiotherapy	January 2021 and January 2023 in two tertiary hospitals	nasopharyngeal carcinoma patients after radiotherapy	NRS 2002 score of > 3	NRS 2002 scale

a: European Society of Clinical Nutrition and Metabolism (ESPEN) Malnutrition Diagnostic Criteria. b: GLIM recommends that the combination of at least one phenotypic criterion and one etiologic criterion is required Weight loss (>5% within past 6 months), reduced BMI (Asia: < 18.5 if age < 70 year, or < 20 if age >70 year), and reduced muscle mass were categorized as phenotypic criteria, and reduced food intake/assimilation and disease burden/inflammation as etiologic criteria.

### Model development and predictors

**Figure 2:**
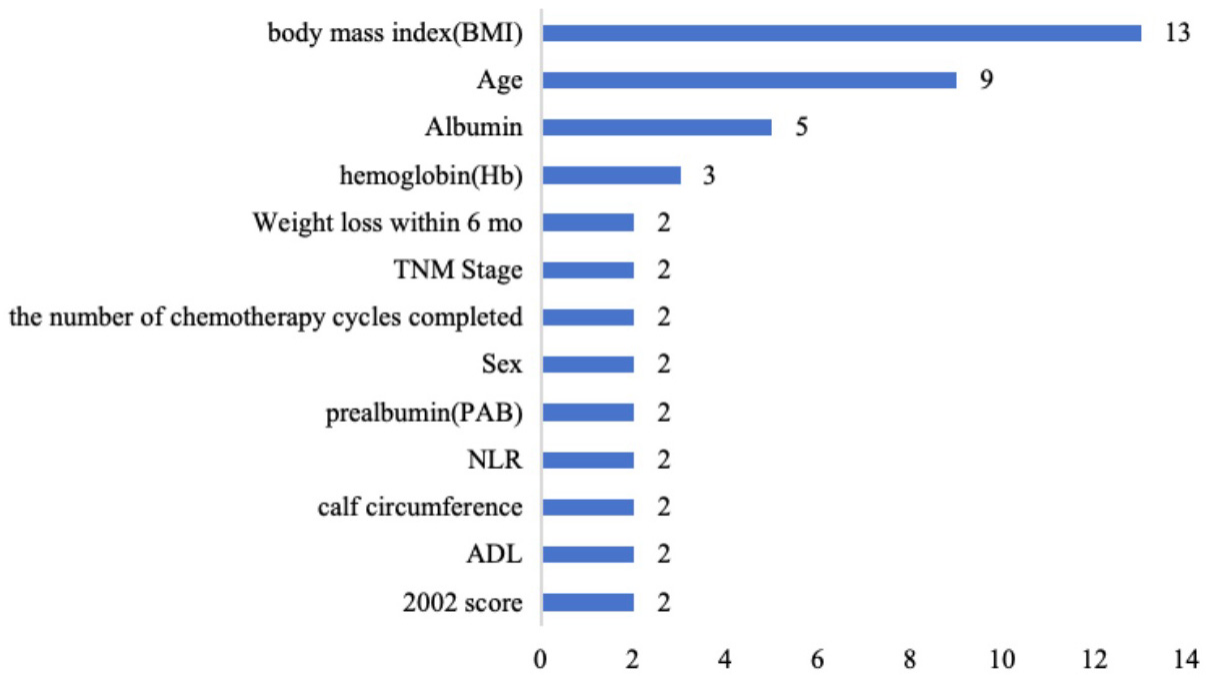
The frequency of common predictors (*n* > 1).

Among the included studies, the sample size ranged from 120 to 4,487 participants. Among the sixteen studies, six studies did not report the specific number of outcome events (20, 22, 24, 25, 28, 29), and three studies used the same dataset (14, 19, 29). The number of candidate predictors ranges from 11 to 36. The methods for selecting predictors include univariate and multivariate analysis, lasso regression, and the “Caret” package. Only three studies reported that all data were included in the analysis (21, 26, 31). thirteen studies all reported data loss during the patient inclusion process (13, 14, 18–20, 22–25, 27–30). Among them, nine studies directly excluded missing cases(13, 14, 18–20, 24, 25, 28, 29), two studies used random forest padding in addition to directly excluding missing cases (22, 23), one study used multiple interpolation (27), and one study adopted corresponding treatment methods based on the type of variable (30). In the optimal model, the number of final predictors ranges from 3 to 10. the most common predictive factors include body mass index (BMI), Age, albumin (ALB), hemoglobin (Hb), weight loss within 6 month, TNM Stage, and the number of chemotherapy cycles completed, sex, prealbumin (PAB), neutrophil-to-lymphocyte ratio (NLR), calf circumference, activities of daily living (ADL), 2002 score. The frequency distribution of the most common predictors (*n* > 1) is shown in Figure 2. The detailed frequency table of all predictors in the model can be found in the Supplementary materials (Table S2). The basic characteristics and final predictors of the model are shown in Table 3.

**Table 3:** Overview of the basic information about prediction models.

References	Development sets/validation sets		Number of candidate predictors	Final predictors number/catalogs	Missing data	
	Sample size	Outcome events			Number	Method
Park et al. (13)	1,281/-	152/-	11	3/Preoperative BMI, Sex, Operation type	Patients unable to undergo curative resection due to their stage IV GC (*n* = 68); Patients with missing weight data at all time-points of follow-up were also excluded (*n* = 95)	Exclude
Yin et al. (14)	914/305	LR: 204/88 CRT: 195/83	36	6/Sex, BMI, Weight loss within 6 Mo, Weight loss beyond 6 Mo, Calf circumference, Handgrip strength/weight ratio	Excluding 17 patients without complete database information and 2 patients without follow-up data	Exclude
Zhang et al. (18)	702/-	490/-	11	4/Age, Tumor type, BMI, PA-LA	427 excluded: multiple hospitalization records; 47 excluded: missing test results of phase angle	Exclude
Yin et al. (19)	2,998/1,000	849/271	28	5/age, weight loss within 6 Mo, BMI, calf circumference, and the Nutritional Risk Screening 2002 score	Excluded 25 patients with non-solid tumors and 2 patients with an unclear pathological diagnosis.	Exclude
Dai et al. (20)	242/102	-/-	34	5/TNM Stage, CFG, PAB, NLR, EN 48 h post-op	39 cases were excluded because the medical records or follow-up records were incomplete, 55 patients were excluded because of their age/severe liver and kidney dysfunction/combined blood or rheumatic system diseases/distant metastasis/died within 3 months	Exclude
Tang et al. (31)	506/-	149/-	23	7/age, BMI, Eastern Cooperative Oncology Group, metastatic disease, albumin, fatigue, change in stool/bowel habits	none	/
Yu et al. (21)	84/36	32/15	12	3/age, odds ratio, ECOG PS	none	/
Duan et al. (22)	360/90	-/-	22	4/ADL, ALB, BMI, age.	317 cases were removed due to repeated hospital admissions, 24 were excluded due to communication barriers (n = 14) or psychiatric conditions (n = 10), and an additional 10 were omitted due to incomplete nutritional status data. the missing data are less than 10 %	Exclude random forest filling
Duan et al. (23)	315/135	147/62	22	4/age, ALB, BMI, ADL	317 cases were removed due to repeated hospital admissions, 24 were excluded due to communication barriers (*n* = 14) or psychiatric conditions (*n* = 10), and an additional 10 were omitted due to incomplete nutritional status data.	Exclude random forest filling
line Huang et al. (24)	218/94	-/-	17	3/BMI, lymphocyte and ALB	Chemoradiotherapy and chemotherapy (*n* = 58), Lack of image data (*n* = 31), Lack of clinical date (*n* = 125), surgery at L3 vertebral level (*n* = 13), Combine with diabetes (*n* = 33)	Exclude
Li et al. (25)	198/86	-/-	21	3/BMI, Hb, Radscore	Malnutrition screening was not performed; distant metastasis occurred; lack complete case records	Exclude
Wang et al. (26)	280/119	172/-	31	6/age, the number of chemotherapy cycles completed, total radiation dose received, BMI, ALB, chloride	none	/
Wang et al. (27)	2,715/672	1,081/288	26	10/age, presence of a digestive system tumor, TNM staging, KPS score, history of alcohol consumption, history of infections, presence of combined thoracic and abdominal fluid, Hb, Cr levels, NLR	209 clinical data missing >20%; 345 bleeding from the digestive tract or vital organs; 410 admitted to ICU or CCU347 life expectancy < 3 months; 34 unclear diagnosis	Exclude multiple imputation
Wang et al. (28)	121/270	-/-	30	4/NRS-2002, prolonged bed rest, BMI, and PNI	336 life expectancy < 6 months 68 severe infections; 49 severe cardiac, pulmonary and renal insufficiency; 169 admitted to ICU or CCU; 108 bleeding from the digestive tract, respiratory tract or liver; 11 clinical data missing >20%	Exclude
Wu et al. (29)	3,365/1,122	-/-	46	4/BMI, age, HGS, MAMC, HDL, triglyceride, Hb, PAB	1998 subjects had incomplete information; 5,590 subjects were lost to follow-up	Exclude
Zhu et al. (30)	258/125	78/39	24	4/BMI, chemotherapy cycles, CVC, ALT	Concurrent Malignancies or Metastatic Diseases (*n* = 11); Significant Medical Conditions or Complications (*n* = 4); Mental illness or cognitively function (*n* = 1); severe malnutrition (*n* = 5)	Exclude imputed using the mean or the median.

PA-LA, phase angle-left arm; MM: moderate malnutrition; SM, severe malnutrition; BMI, body mass index; TNM Stage, Tumor node metastasis stage; CFG, cardiac function grading; PAB, prealbumin; NLR: neutrophil-to-lymphocyte ratio; EN 48 h post-op: enteral nutrition within 48 h post- operation; Hb, hemoglobin; Radscore, radiomics characteristic score; ADL, Activities of Daily Living; ALB, Albumin; CVC, central venous catheter; ALT, alanine aminotransferase.

### Model performance and presentation

A total of 42 predictive models were developed from the 16 included studies. A prediction model for malnutrition in cancer patients was established by using methods such as logistic regression (LR), deep learning (DL), decision tree (DT), classification regression tree (CART), extreme gradient boosting (XGBoost) algorithm, random forest (RF), multilayer Perceptron (MLP), and support vector machine (SVM). Among them, fifteen studies used Logistic regression to establish models (13, 14, 18, 20–31). The area value under the curve (AUC) (ranging from 0.745 to 1.000 in the development set and from 0.687 to 0.982 in the validation set) was often reported as an indicator of the degree of model differentiation. In addition, accuracy, sensitivity and specificity were also commonly used discriminant indicators for models. A study developed the best-performing model using random forest (29). The area value under the curve (AUC) was 1.000 (95%CI: 1.000–1.000), the accuracy, sensitivity and specificity were all 1.000. Eleven studies reported the degree of calibration using the Hosmer-Lemeshow test, calibration curves or Brier score. Seven studies reported the cut-off value (18, 22, 24–26, 28, 30). In terms of model validation, fourteen studies employed internal validation (13, 14, 19–30), among which eight studies used random split validation (20–27), six studies employed the bootstrap method (13, 14, 20, 27, 28, 30), and five studies employed k-fold validation (5- fold or 10- fold) (19, 21, 24, 25, 29). However, only one study reported external validation in detail (30). The presentation forms of predictive models included risk scoring, nomograms, tree diagrams, web prediction tools, etc. The nomogram was the most commonly used method for model display. The performance and representation of the prediction model are shown in Table 4.

**Table 4:** Performance and presentation of malnutrition prediction models in cancer patients.

References	Method (Num.)	Discrimination (95 % CI)		Calibration	Cut-off value	Internal/External validation	Representation form
		Development sets	Validation sets				
Park et al. (13)	LR (1)	C-index: 0.91(0.89, 0.94)	/	Calibration plot	/	Bootstrap/-	Nomogram, prediction scoring table
Yin et al. (14)	LR, CRAT (2)	AUC LR: 0.974 (0.962, 0.985) DT: 0.978 (0.964,0.992)	AUC LR: 0.982 (0.969, 0.995) DT: 0.969 (0.945, 0.994)	LR: Hosmer-Lemeshow test, calibration curve DT: none	/	bootstrap/-	Nomogram, tree diagrams
Zhang et al. (18)	LR (1)	AUC: 0.813 sensitivity: 0.759 Specificity: 0.733	/	/	0.29	-/-	tree diagrams
Yin et al. (19)	CART (1)	AUC: 0.963 accuracy: 0.950	AUC: 0.964 accuracy: 0.955	/	/	Cross-Validation/-	tree diagrams
Dai et al. (20)	LR (1)	AUC: 0.840 (0.787, 0.884) Youden index: 0.536	AUC: 0.854 (0.770, 0.916)	calibration curve, Hosmer-Lemeshow test	/	random split: 7:3 Bootstrap/-	nomogram
Tang et al. (31)	LR (1)	AUC:0.745 (0.697–0.793)	/	Hosmer-Lemeshow test	/	-/-	nomogram
Yu et al. (21)	LR (3)	AUC: radiomics model: 0.778 (0.339–1.000) clinical model: 0.847 (0.577–1.000) combined predictive model: 0.972 (0.895–1.000)	AUC: radiomics model: 0.776 (0.623–0.930) clinical model: 0.776 (0.607–0.946) combined predictive model: 0.805 (0.713–0.996)	Calibration curves	/	random split: 7:3 10-fold cross-validation/-	nomogram
Duan et al. (22)	XGBoost, LR, RF, AdaBoost, GNB, CNB, MLP, SVM, KNN (9)	best model: XGBoost model AUC: 0.984 accuracy: 0.945 sensitivity: 0.967	best model: XGBoost model AUC: 0.945 accuracy: 0.872 sensitivity: 0.968	calibration curve	0.601	random split: 8:2/-	The web-based user-friendly calculator
Duan et al. (23)	LR (1)	AUC: 0.905	AUC: 0.830	calibration curve	/	random split: 7:3/-	user-friendly web-based tool
Huang et al. (24)	LR, RF, SVM, NaiveBayes, KNN, ExtraTrees, XGBoost, LightGBM, GradientBoosting, MLP, AdaBoost (11)	Multilayer Perceptron model: AUC: 0.806 (0.7485–0.8635) accuracy: 0.734 mixed model: AUC: 0.909 (0.869-0.948) accuracy: 0.845 specificity: 0.883 sensitivity: 0.796	Multilayer Perceptron model: AUC: 0.769 (0.673–0.863) accuracy: 0.766 specificity: 0.680 sensitivity: 0.795 mixed model: AUC: 0.857 (0.782–0.931) accuracy: 0.861 specificity: 0.780 sensitivity: 0.818	Calibration curve	0.506/0.435	random split: 7:3 5-fold cross-validation/-	nomogram
Li et al. (25)	LR (1)	AUC: 0.895 (0.844–0.945) accuracy: 0.957 sensitivity: 0.718	AUC: 0.880 (0.806-0.954) accuracy: 0.930 sensitivity: 0.698	Hosmer-Lemeshow test, calibration curve	0.651/0.655	random split: 7:3 10-fold cross-validation/-	nomogram
Wang et al. (26)	LR (1)	AUC: 0.868 (0.822–0.905)	AUC: 0.863 (0.788–0.919)	Hosmer-Lemeshow test, Brier scores	0.6	random split: 7:3/-	nomogram
Wang et al. (27)	LR (1)	AUC: 0.793 (0.776–0.810)	AUC: 0.832 (0.801–0.863)	Calibration curves, Brier scores, Hosmer–Lemeshow test	/	random split: 8:2 Bootstrap/-	nomogram
Wang et al. (28)	LR (1)	AUC: 0.958 (0.937–0.979) sensitivity: 0.843 specificity: 0.937	/	/	0.78	Bootstrap/-	nomogram
Wu et al. (29)	DT, ADA, RF, SVM, LR, NNET (6)	best model: RF AUC: 1.000 (1.000–1.000) accuracy: 1.000 sensitivity: 1.000 specificity: 1.000	best model: RF AUC: 0.830 (0.805–0.854) accuracy: 0.775 sensitivity: 0.835 specificity: 0.742	/	/	10-fold cross-validation/-	online predic- tion tool
Zhu et al. (30)	LR (1)	AUC: 0.786 (0.722–0.851) sensitivity:0.701 specificity:0.838 Youden index: 0.539	AUC: 0.687 (0.581–0.793)	Hosmer–Lemeshow test, Calibration curves	0.453	Bootstrap/geographical validation	dynamic online nomogram

CI, confidence interval; DCA, Decision curve analysis; AUC, area under curve; XGBoost, extreme gradient boosting; LR, logistic regression; RF, random forest; ADA, adaptive boost machine; DT, decision tree; NNET, neural network; SVM, support vector machine; MLP, Multilayer Perceptron; KNN, K-nearest neighbors; CNB, complement nb; GBN, AdaBoost, Gaussian Nb; CART, classification and regression trees.

### Risk of bias and applicability

**Figure 3:**
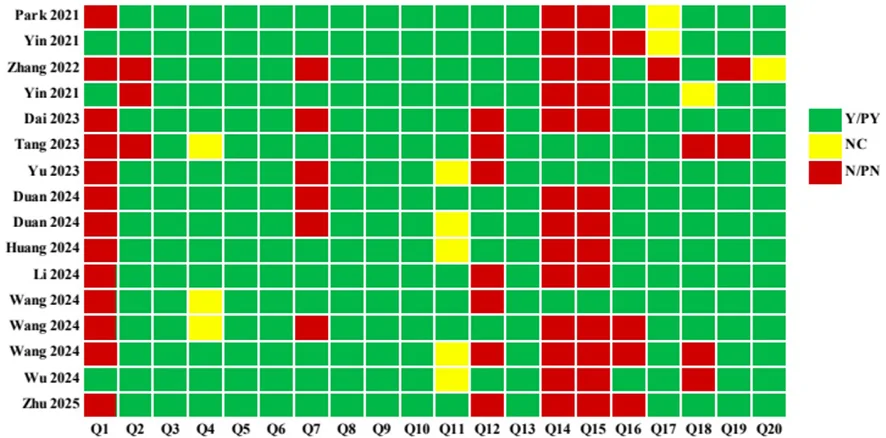
Risk of bias assessment by using PROBAST across 20 signaling questions.

**Figure 4:**
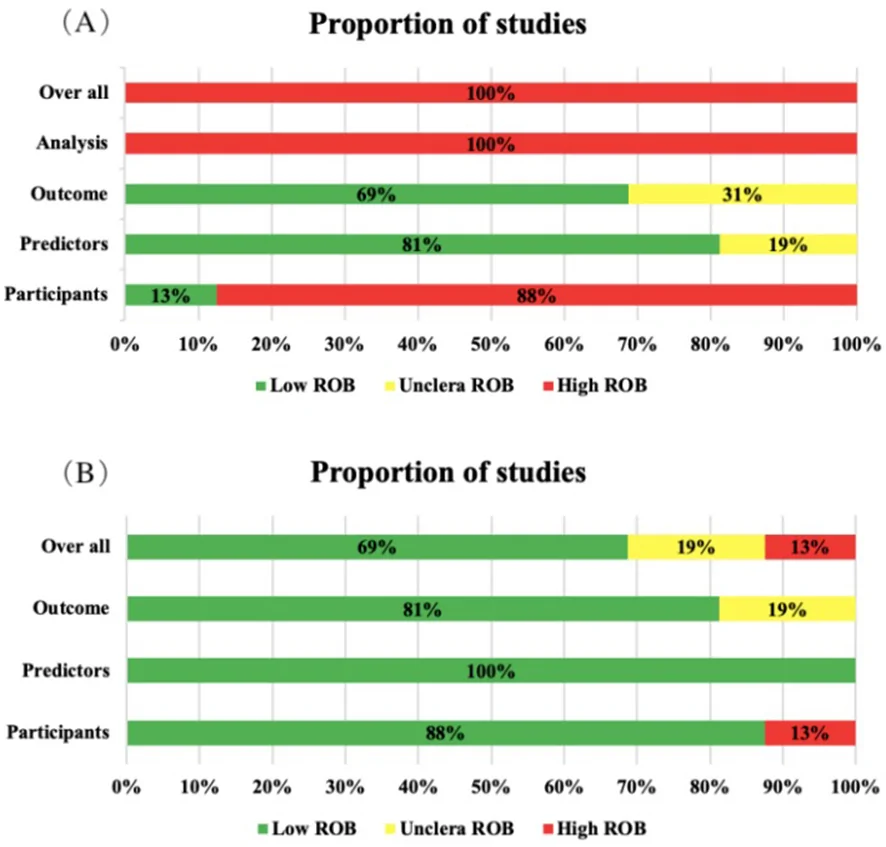
Results of Bias and applicability assessment of 16 studies. **(A)** Overall rate of risk of bias (ROB) based on PROBAST: low, green; high, red; unclear, yellow. **(B)** Overall rate of applicability based on PROBAST: low, green; high, red; unclear, yellow.

The results of the risk of bias and applicability evaluation using the PROBAST are summarized in Table 5, and Figures 3, 4.

**Table 5:** Results of bias and applicability risk assessment according to PROBAST.

References	Risk of bias			Applicability			Overall		
	Participants	Predictors	Outcome	Analysis	Participants	Predictors	Outcome	Risk of bias	Applicability
Park et al. (13)	–	+	+	–	+	+	+	–	+
Yin et al. (14)	+	+	+	–	+	+	+	–	+
Zhang et al. (18)	–	+	+	–	–	+	+	–	–
Yin et al. (19)	–	+	+	–	–	+	+	–	–
Dai et al. (20)	–	+	+	–	+	+	+	–	+
Tang et al. (31)	–	?	+	–	+	+	+	–	+
Yu et al. (21)	–	+	?	–	+	+	+	–	+
Duan et al. (22)	–	+	+	–	+	+	+	–	+
Duan et al. (23)	–	+	?	–	+	+	+	–	+
Huang et al. (24)	–	+	?	–	+	+	+	–	+
Li et al. (25)	–	+	+	–	+	+	+	–	+
Wang et al. (26)	–	?	+	–	+	+	?	–	?
Wang et al. (27)	–	?	+	–	+	+	+	–	+
Wang et al. (28)	–	+	?	–	+	+	?	–	?
Wu et al. (29)	+	+	?	–	+	+	?	–	?
Zhu et al. (30)	–	+	+	–	+	+	+	–	+

+: low risk of bias; -: high risk of bias; ?: unclear risk of bias.

#### Participants

In the field of participants, fourteen studies were identified as having a high risk of bias (13, 18–28, 30, 31). Of these, thirteen studies (13, 18, 20–28, 30, 31) were found to have selection bias due to their retrospective or cross-sectional study designs, meaning that their sampling strategies were unlikely to accurately represent the target population. Furthermore, in one study (19), 25 patients with non-solid tumors were excluded, which contradicts the study's population for cancer patients. Therefore, there may be sampling bias that leads to an inconsistent range of the target population.

#### Predictors

In the predictive domain, three studies (26, 27, 31) were unclear. Three studies adopted a cross-sectional design. It was not clear whether the results of the indicator tests were interpreted without knowledge of the reference standard results, leading to an unclear risk of bias. The remaining studies all reported standardized procedures for patient assessment, data collection, and documentation of the predictor variables.

#### Outcome

In the outcome domain, six studies (18, 20–23, 27) were at high risk of bias and three studies (24, 28, 29)were unclear. Six studies did not use a unified international standard to define malnutrition. These studies relied on self-reported measurements. However, due to the subjectivity of patients, the assessment might not be very reliable. Three studies did not report the time interval between predictor assessment and outcome determination, and thus the risk of bias was rated as unclear.

#### Analysis

In the field of analysis, all studies were rated as high-risk bias. Among them, seven studies (20, 21, 25, 26, 28, 30, 31) did not meet the recommended number of events (EPV) of ≥20 per candidate variable (33). In thirteen studies (13, 14, 18–20, 22–25, 27–30), the missing data were handled improperly, resulting in the direct exclusion of patients due to missing information or inability to follow up. In addition, two studies (22, 23)used the random forest filling method, one study (30) selected the processing method based on the variable type, and one study (25) did not report the number of missing values. Twelve studies (13, 14, 18–21, 24, 26–28, 30, 31) avoided selecting variables based on univariate analysis. None of the included studies provided any information regarding the complexity of the data. Twelve studies (13, 14, 20–27, 30, 31) fully reported the differentiation and calibration of predictive models, fourteen studies (13, 14, 19–30) reported internal validation of the models, and one study (30) conducted external validation. However, no form of validation was found in two studies (18, 31), and three studies (22, 23, 26) did not consider overfitting, underfitting, or performance optimism.

#### Applicability

In terms of applicability, two studies (18, 19) were considered to have a high risk of bias, while the risk of bias for three studies (26, 28, 29) was unclear. In the field of participants, two studies involved participants who did not align with the review's research question and were therefore rated as having a high risk of bias. All studies in the prediction domain were evaluated as low-risk. In the outcome domain, three studies did not report information related to predicting the duration of malnutrition, and these studies were rated as unclear.

## Discussion

This systematic review comprehensively sorted out the predictive models for malnutrition in cancer patients for the first time. A total of 16 studies were screened out, covering 42 models. Their characteristics and performances were systematically summarized, and the risks of bias and applicability were evaluated. These models generally demonstrated moderate to excellent predictive performance, and we also summarized the most frequently occurring predictor variables. However, all studies were rated as having a high overall risk of bias, and two of them also had a high risk of applicability, mainly due to the unclear selection of subjects and the description of clinical scenarios. The above results will provide an important basis for the subsequent development, validation, update and implementation of cancer malnutrition prediction models. However, before it is officially put into clinical practice, more in-depth evaluations are still needed.

Among all the studies, BMI and serum albumin are reliable predictors of malnutrition in patients with various tumors. These predictors provide valuable guidance for future research on determining candidate factors. In addition, dynamic monitoring and assessment of cancer patients based on these predictive factors enable healthcare professionals to effectively identify and reduce the incidence of malnutrition at an early stage, and provide nutritional support for cancer patients. Nutritional support for cancer patients primarily includes enteral nutrition, parenteral nutrition or oral nutritional supplements, and nutrients include ω-3 polyunsaturated fatty acids, branched-chain amino acids, L-glutamine, L-L-carnitine, β-hydroxyl -β-methyl butyric acid, etc. (34–36) Healthcare professionals can provide early, personalized and targeted nutritional support and health guidance to patients with different types of cancer. For instance, patients with gastrointestinal cancers experience a higher incidence and greater severity of malnutrition due to reduced food intake and absorption during the perioperative period (37). Combined with the tumor's own nutritional demands, patients with gastrointestinal cancer may benefit from preoperative nutritional support to mitigate the systemic effects of malnutrition. In addition, previous studies have shown that approximately 20% to 70% of cancer patients experience chemotherapy-induced nausea and vomiting during or after chemotherapy (38), due to the influence of chemotherapy-induced symptom clusters such as nausea and vomiting, changes in taste, and decreased appetite, the absorption and intake of nutrients by cancer patients undergoing chemotherapy are also at a relatively low level. Therefore, healthcare professionals must first prevent or alleviate a series of adverse drug reactions—such as nausea and vomiting—caused by chemotherapy (39), for instance, providing dietary consultation to cancer patients in combination with personalized diets based on specific nutrient intake (40), or guiding cancer patients to adopt a diet behavior of eating small meals frequently, chewing food slowly and thoroughly, and restricting the intake of specific foods (avoiding the intake of processed meats) (41). Therefore, nutritional support for cancer patients' needs to be tailored and adapted according to the disease condition of each individual patient.

Given the differences in study populations, designs, the predictors included, and the methods used to assess these predictors, there are both similarities and differences among the predictors in various study. For example, treatment approaches differ among patients with varying tumor types and origins, and the predictors of malnutrition also differ between patients undergoing surgery and those receiving chemotherapy. Compared with patients receiving chemotherapy, malnutrition in cancer patients undergoing surgery develops malnutrition earlier than those receiving chemotherapy (37). However, as the number of chemotherapy sessions and stage of treatment progress, malnutrition in cancer patients receiving chemotherapy becomes more difficult to correct. BMI is an easily accessible predictor in routine clinical practice and is the most commonly used indicator for predicting malnutrition in research. However, cancer patients generally show weight loss before diagnosis (42). Nevertheless, only one study (31) assessed malnutrition at the time of diagnosis, which is of great significance for the early identification of the nutritional status of cancer patients. Furthermore, the studies by Park et al. (13) and Dai et al. (20) focused on patients after radical gastrectomy for gastric cancer, while Huang et al. (24) and Li et al. (25) focused on patients with gastric cancer before surgery. Although all studies involved gastric cancer patients, differences in treatment stage, cancer stage, and histological type may limit the practicality and clinical applicability of these models in a broader population. Therefore, future research should conduct precise assessments and stratification of patients' nutritional status by tumor type at the time of diagnosis, and based on this, construct dynamically updated, individualized malnutrition risk prediction models to track the onset and progression of malnutrition. With the help of these stratified models, healthcare professionals can formulate targeted nutritional intervention plans to alleviate complications caused by malnutrition and comprehensively improve the quality of life of cancer patients.

Another notable issue is that there is significant heterogeneity in the measurement methods and determination criteria for malnutrition in cancer patients. In six studies, the determination of malnutrition in cancer patients was based on the Patient Subjective Global Assessment (PGSGA) (18, 20–23, 26). This scale is completed through a combination of patient self-assessment and healthcare provider evaluation. The patient self-assessment section includes modules on weight changes, food intake, symptoms, and activity and function over the past six months. Due to individual differences in reporting standards, retrospective or recall biases are likely to occur (43). furthermore, different studies have different operational definitions of the scoring rules for PGSGA. One study determined that the score for malnutrition is greater than or equal to two points (26), while the remaining five studies determined it to be greater than or equal to four points (18, 20–23). The application of predictive models may lead to different results due to different classification levels, which may cause cancer patients to have different understandings of their own nutritional status, thereby affecting their compliance with treatment or intervention. In addition, the differences in classification levels make it difficult to compare data across studies or populations. Six studies (14, 19, 24, 27–29) evaluated malnutrition in cancer patients based on the Global Leadership on Malnutrition Initiative (GLIM), but two of them (24, 29) only used phenotypes to determine the occurrence of malnutrition due to the characteristics of the study population. The impact of digestive tract tumors on diet is objectively present, however, we cannot deny the existence of individual differences (44). Therefore, inappropriate assessment methods may increase the risk of missed diagnosis or misdiagnosis of malnutrition. It is suggested that future research should adhere to the measurement tools, judgment criteria and classification levels recommended in the guidelines, uniformly formulate the assessment content and operational definitions of malnutrition in tumor patients, and establish accurate predictive models to identify high-risk groups.

Furthermore, although some models demonstrate moderate to good predictive performance, all studies are rated as having a high risk of bias. The most prominent sources of bias include retrospective study designs, insufficient sample sizes, inappropriate reporting and handling of missing data, and a lack of internal and external validation. The high risk of bias suggests that the development methods, processes and clinical applicability of the malnutrition risk model for cancer patients need to be further improved. Therefore, it is recommended that future research designs adopt prospective cohort designs or randomized controlled trials to reduce sampling bias and the influence of confounding variables on results, thereby enhancing the models' ability to draw causal inferences. Furthermore, an adequate sample size is crucial for reducing overfitting and evaluating the stability of the model's predictive ability. According to the recommended PROBAST guidelines, when the number of events for each variable is at least 20, the model has a low optimism bias (12). It is worth noting that thirteen studies reported the existence of missing data in the research population (13, 14, 18–20, 22–25, 27–30), but only four studies conducted analysis methods other than direct deletion of data (22, 23, 27, 30). For example, one study (27) adopted multiple interpolation methods to handle the missing data, enhancing the credibility of the report results in accurately reflecting these data. One study (25) only reported the causes of data missing without providing specific values. We suggest that researchers provide a complete report on the causes and quantities of data missing to ensure the robustness of the model. In addition, internal and external validation can evaluate the performance of the model, correct model overfitting, and assess the generalization ability and clinical usability of the model, which is crucial for the development of predictive models (45, 46). However, only one study reported both external and internal validation simultaneously (30), while the remaining fourteen studies only reported internal validation (13, 14, 19–30), indicating that the validation of malnutrition prediction models for tumor patients is insufficient, which is consistent with Tugwell et al. (46) Future researchers developing, updating or validating risk prediction models for malnutrition in cancer patients may refer to the PROBAST (12) and Transparent Reporting of a Multivariable Prediction Model for Individual Prognosis or Diagnosis (TRIPOD) (47) checklists to enhance applicability and reduce the risk of bias.

Traditional logistic regression is the most commonly used method for developing predictive models. In addition, radiomics and machine learning algorithms offer new methodological perspectives for developing models to predict malnutrition in cancer patients. Radiomics can help healthcare professionals explore more valuable clinical predictors and provide higher efficiency for the diagnosis of malnutrition. Algorithms such as random forests, decision trees, and deep learning can help researchers efficiently manage complex and multi-dimensional datasets and enhance the reliability, performance, predictability, and accuracy of malnutrition risk models (48). Five studies (14, 20, 22, 24, 29) constructed oncology malnutrition prediction models using machine learning algorithms, which were capable of capturing complex patterns and variable interactions that were difficult to be clearly observed in traditional logistic regression (49). However, machine learning generally faces the risk of overfitting, a research report by Wu et al. (29) indicates that the area under the curve for the development set reaches 1.00, whereas it is 0.830 for the validation set; this suggests that the model constructed by Wu et al. may exhibit overfitting, models perform exceptionally well on the training set but may not be applicable to external data. Therefore, it is essential to employ rigorous validation strategies, such as cross-validation and testing on independent external datasets, to minimize this risk as much as possible. Subsequent multi-center and large-sample studies are still needed to systematically evaluate the extrapolation, interpretability and potential bias of these models, laying a solid foundation for their true application in clinical practice.

The review empowers medical staff to shift from reactive to proactive in malnutrition management. By applying easy-to-use predictors and validated models, medical staff can rapidly identify high-risk patients at different treatment stages. Consequently, individualized nutritional support plans—such as dietary counseling, frequent small meals, and enteral or parenteral nutrition—can be initiated early, potentially reducing chemotherapy-induced nausea and vomiting, postoperative complications, and overall treatment interruptions. The risk of malnutrition among cancer patients varies depending on the type of cancer, the stage of treatment, and individual patient characteristics. This supports the development of a more detailed and dynamic framework to understand the issue of malnutrition in cancer treatment - moving away from static risk factors and toward predictive models with hierarchical structures and specific time dimensions. Incorporating the concepts of scientific and predictive analysis into this framework could further enrich the theoretical model of supportive care.

Our research has several limitations. Firstly, we only include studies published in English, which might lead to publication bias and omit some potential research. Secondly, most of the models are developed and verified in China, which has geographical limitations. Furthermore, most of the research types are retrospective studies, which to some extent leads to significant deviations in the predictors. It is worth noting that due to the significant heterogeneity of the included studies, we are unable to quantitatively analyze the predictive performance of the malnutrition prediction model for cancer patients, which limit the applicability and comparability of the research models. Therefore, future research can consider conducting multi-center and large-sample applied studies to develop risk prediction models with repeatability and high prediction accuracy, in order to identify the occurrence of malnutrition at an early stage. In addition, with the rapid development of the field of artificial intelligence, data mining can be considered. By integrating predictive models with related software and algorithms, efficient and convenient assessment software can be developed to meet the needs of screening for malnutrition in cancer patients in different clinical scenarios.

## Conclusions

This systematic review evaluates 16 studies that developed 42 predictive models for malnutrition in cancer patients. According to the assessment of the PROBAST tool, all models show a high risk of bias, and two studies have raised concerns about their applicability. The current malnutrition risk prediction models still lack clinical applicability. Therefore, in the future, strict and standardized risk prediction model construction should be carried out in accordance with the recommendations of the CHARMS and TRIPOD lists, and rigorous internal and external validation should be conducted to reduce the risk of bias and develop prediction models for malnutrition in cancer patients suitable with clinical practice.

## Data Availability

The original contributions presented in the study are included in the article/Supplementary material, further inquiries can be directed to the corresponding author.
