# Supplementary material for: Risk prediction models for malnutrition in patients with cancer: a systematic review
Source: Front Nutr. 2026 Aug 6;13:1900944. doi: 10.3389/fnut.2026.1900944 (PMC13492061; doi:10.3389/fnut.2026.1900944)
Supplement: Supplementary file 1 [file Supplementary_File_1.docx]

Table S1 Database search results

| **Item** | **Database** | **Formula** | **Number** |
| --- | --- | --- | --- |
| 1 | PubMed | (((predicti* model[Title/Abstract]) OR (prediction[Title/Abstract] OR risk model[Title/Abstract] OR risk assessment[Title/Abstract] OR risk prediction[Title/Abstract] OR risk score[Title/Abstract] OR nomogram[Title/Abstract] OR forecast model[Title/Abstract] OR diagnostic model[Title/Abstract] OR prediction tool[Title/Abstract])) AND ((((Neoplasms[MeSH Terms]) OR (Neoplas*[Title/Abstract] OR Malignanc*[Title/Abstract])) OR (tumor[Title/Abstract] OR cancer[Title/Abstract] OR malignant[Title/Abstract] OR carcinoma[Title/Abstract] OR oncology[Title/Abstract])) OR (neoplasms[Title/Abstract]))) AND (((((malnutrition[MeSH Terms]) OR (undernourishment*[Title/Abstract] OR Nutritional Deficienc*[Title/Abstract] OR dystroph*[Title/Abstract])) OR (undernutrition[Title/Abstract] OR hyponutrition[Title/Abstract] OR paratrophy[Title/Abstract] OR hypotrophy[Title/Abstract]))) OR (malnutrition[Title/Abstract])) | 423 |
| 2 | EBSCO | (SU (predicti* model OR prediction OR risk model OR risk assessment OR risk prediction OR risk score OR nomogram OR prediction model OR forecast model OR predictive model OR diagnostic model OR prediction tool)) AND (MM "Neoplasms+" OR SU (Neoplas* OR Malignanc*) OR SU (neoplasms OR neoplasia OR tumor OR cancer OR malignant OR carcinoma OR oncology)) AND (MM "Malnutrition+" OR SU (undernourishment* OR Nutritional Deficienc* OR dystroph* OR malnutrition OR undernutrition OR undernourishment OR dystrophia OR dystrophy OR hyponutrition OR paratrophy OR hypotrophy)) | 103 |
| 3 | Medline | neoplasms OR neoplasia OR tumor OR cancer OR malignant OR carcinoma OR oncology OR Neoplas* OR Malignanc* (Topic) AND malnutrition OR undernutrition OR undernourishment OR dystrophia OR dystrophy OR hypernutrition OR panatrophy OR hypotrophy OR undernourishment* OR Nutritional Deficienc* OR dystroph* (Topic) AND predicti* model OR prediction OR risk model OR risk assessment OR risk prediction OR risk score OR nomogram OR prediction model OR forecast model OR predictive model OR diagnostic model OR prediction tool (Topic) and English (Languages) | 2676 |
| 4 | Web of science | neoplasms OR neoplasia OR tumor OR cancer OR malignant OR carcinoma OR oncology OR Neoplas* OR Malignanc* (Topic) and malnutrition OR undernutrition OR undernourishment OR dystrophia OR dystrophy OR hypernutrition OR panatrophy OR hypotrophy OR undernourishment* OR Nutritional Deficienc* OR dystroph* (Topic) and predicti* model OR prediction OR risk model OR risk assessment OR risk prediction OR risk score OR nomogram OR prediction model OR forecast model OR predictive model OR diagnostic model OR prediction tool (Topic) and English (Languages) | 2705 |
| 5 | Scopus | TITLE-ABS-KEY: ((“Neoplas*” OR “Malignanc*” OR “neoplasms” OR “neoplasia” OR “tumor” OR “cancer” OR “malignant” OR “carcinoma” OR “oncology”) AND (“undernourishment*” OR “Nutritional Deficienc*” OR “dystroph*” OR “malnutrition” OR “undernutrition” OR “undernourishment” OR “dystrophia” OR “dystrophy” OR “hyponutrition” OR “paratrophy” OR “hypotrophy”) AND (“predicti* model” OR “prediction” OR “risk model” OR “risk assessment” OR “risk prediction” OR “risk score” OR “nomogram” OR “prediction model” OR “forecast model” OR “predictive model” OR “diagnostic model” OR “prediction tool”)) | 122 |
| 6 | Cochrane Library | #1 MeSH descriptor: (neoplasms) explode all trees  #2 MeSH descriptor: (Malnutrition) explode all trees  #3 (neoplasms OR neoplasia OR tumor OR cancer OR malignant OR carcinoma OR oncology OR Neoplas* OR Malignanc*):ti.ab.kw  #4 (malnutrition OR undernutrition OR undernourishment OR dystrophia OR dystrophy OR hypernutrition OR panatrophy OR hypotrophy OR undernourishment' S -  OR Nutritional Deficienc* OR dystroph*):ti.ab.kw  #5 (predicti* model OR prediction OR risk model OR risk assessment OR risk prediction OR risk score OR nomogram OR prediction model OR forecast model OR predictive model OR diagnostic model OR prediction tool):ti,ab.kw  #6 #1 OR #3  #7 #2 OR #4  #8 #5 AND #6 AND #7 | 314 |
