# Supplementary material for: Risk prediction models for malnutrition in patients with cancer: a systematic review
Source: Front Nutr. 2026 Aug 6;13:1900944. doi: 10.3389/fnut.2026.1900944 (PMC13492061; doi:10.3389/fnut.2026.1900944)
Supplement: Supplementary file 2 [file Supplementary_File_2.docx]

Table S2 The frequency table of all final predictors.

| Rank | Predictors | Frequency |
| --- | --- | --- |
| 1 | body mass index (BMI) | 13 |
| 2 | Age | 9 |
| 3 | Albumin | 5 |
| 4 | Hemoglobin (Hb) | 3 |
| 5 | Weight loss within 6 Mo | 2 |
| 6 | TNM Stage | 2 |
| 7 | the number of chemotherapy cycles completed | 2 |
| 8 | Sex | 2 |
| 9 | prealbumin (PAB) | 2 |
| 10 | NLR | 2 |
| 11 | calf circumference | 2 |
| 12 | ADL | 2 |
| 13 | 2002 score | 2 |
| 14 | Weight loss beyond 6 Mo | 1 |
| 15 | Tumor type | 1 |
| 16 | triglyceride | 1 |
| 17 | total radiation dose received | 1 |
| 18 | Radscore | 1 |
| 19 | prolonged bed rest | 1 |
| 20 | presence of combined thoracic and abdominal fluid | 1 |
| 21 | presence of a digestive system tumor | 1 |
| 22 | PNI | 1 |
| 23 | PA-LA | 1 |
| 24 | Operation type | 1 |
| 25 | odds ratio | 1 |
| 26 | metastatic disease | 1 |
| 27 | MAMC | 1 |
| 28 | lymphocyte | 1 |
| 29 | KPS score | 1 |
| 30 | history of infections | 1 |
| 31 | history of alcohol consumption | 1 |
| 32 | HGS | 1 |
| 33 | HDL | 1 |
| 34 | Handgrip strength/weight ratio | 1 |
| 35 | fatigue | 1 |
| 36 | enteral nutrition within 48 hours post- operation (EN 48 h post-op) | 1 |
| 37 | ECOG PS | 1 |
| 38 | Eastern Cooperative Oncology Group | 1 |
| 39 | Cr levels | 1 |
| 40 | Chloride | 1 |
| 41 | change in stool/bowel habits | 1 |
| 42 | central venous catheter (CVC) | 1 |
| 43 | cardiac function grading (CFG) | 1 |
| 44 | alanine aminotransferase (ALT) | 1 |
